# Myasthenia gravis and autoimmune overlap: Prognostic insight

**DOI:** 10.1371/journal.pone.0334434

**Published:** 2025-10-23

**Authors:** Emel Oguz-Akarsu, Sarra El hamida Lazrak, Deniz Sigirli, Gizem Gullu, Yasemin Dinç, Furkan Saridas, Necdet Karli

**Affiliations:** 1 Department of Neurology, Uludag School of Medicine, Bursa Uludag University, Bursa, Turkey; 2 Department of Statistics and Business Analytics, United Arab Emirates University, Al Ain, UAE; 3 Department of Biostatistics, Faculty of Medicine, Bursa Uludağ University, Bursa, Türkiye; Cleveland Clinic, UNITED STATES OF AMERICA

## Abstract

**Background:**

Autoimmune diseases (ADs) frequently coexist with myasthenia gravis (MG), suggesting shared genetic and immunological mechanisms. However, the impact of comorbid ADs on MG prognosis remains unclear. This study aimed to investigate the prevalence, clinical characteristics, and prognosis of MG patients with comorbid ADs in a Turkish cohort.

**Methods:**

We retrospectively analyzed 302 MG patients treated at a tertiary center between 2010 and 2024. Patients were grouped based on the presence of comorbid ADs. Clinical characteristics, disease severity, treatment response, and prognosis were compared.

**Results:**

Among 302 MG patients, 41 (13.6%) had at least one comorbid AD, with autoimmune thyroid disease (AITD) being the most common (10.6%). ADs were more frequent in females. Patients with and without comorbid ADs showed no significant differences in MG severity, thymectomy rates, myasthenic exacerbations, or overall outcomes (p > 0.05). However, female patients with ADs experienced more frequent myasthenic exacerbations and had a higher rate of rituximab use (p < 0.05).

**Conclusions:**

Comorbid ADs do not significantly impact MG severity or prognosis. Female MG patients with ADs have a higher frequency of myasthenic exacerbations and rituximab use, warranting further research. Early identification of ADs remains essential for optimal patient management.

## 1. Introduction

Autoimmune diseases are a diverse group of conditions where the body’s immune system mistakenly targets its own organs due to immune intolerance to self-antigens. Environmental influences, hormonal changes, genetics, and various immunological variations contribute to the increased susceptibility of individuals with one autoimmune disease to develop additional ones [1]. Studies indicate that the annual incidence of new autoimmune disease cases is as high as 80 per 100,000 people, with a higher prevalence in women than men [2,3].

Myasthenia gravis (MG) is a chronic autoimmune neuromuscular disease characterized by fluctuating muscle weakness and fatigue [4]. While often considered an isolated condition, MG frequently co-occurs with other autoimmune diseases (ADs), and this overlap suggests shared genetic and immunological mechanisms underlying these disorders [5]. Several studies have reported a significant association between MG and other ADs, with autoimmune thyroid disease being the most common comorbidity [6,7]. However, despite numerous studies, the clinical characteristics and prognosis of MG patients with coexisting autoimmune diseases are not fully elucidated.

We analyzed the prevalence of comorbid ADs in a Turkish cohort of MG patients. Additionally, we examined the clinical characteristics and the impact of these comorbid ADs on the prognosis of MG.

## 2. Methods

We retrospectively reviewed all patients with MG admitted to Uludag University Faculty of Medicine Hospital between 2010 and 2024. This hospital is a tertiary university hospital located in Bursa, Turkey, and serves as a major referral center for the South Marmara region. Bursa, the largest city in the region, broadly reflects the demographic characteristics of the South Marmara population. The first data for research purposes was accessed on 02/01/2024.

Inclusion criteria included fluctuating muscle weakness along with the following findings:

(1) positive results on the acetylcholine receptor (AChR) or muscle-specific kinase (MuSK) antibody assay;
(2) more than a 10% decrease in the compound muscle action potential amplitude during repetitive nerve stimulation;
(3) increased jitter on single-fiber electromyography (SFEMG);
(4) a positive response to edrophonium chloride (Tensilon test)

Conditions mimicking MG were excluded. Patients with incomplete data were also excluded from the study. All patients were initially tested for anti-AChR antibodies. Anti-MuSK antibody testing was performed in patients who tested negative for anti-AChR.

The ADs were identified based on previously established criteria, encompassing 81 ADs [8]. Diagnoses of ADs were confirmed through clinical manifestations, laboratory tests (including specific antibody assays), and diagnostic criteria established by relevant specialists.

Clinical data were collected from the recruited patients, including age at disease onset, sex, thymoma concurrence, history of thymectomy, serum antibody status, history of myasthenic crisis, and family history of ADs. Additionally, Myasthenia Gravis Foundation of America (MGFA) classification at onset, the worst MGFA classification during the disease course, overall outcomes, and the presence of other comorbid ADs were recorded.

According to the MGFA-PIS, a favorable outcome was defined as achieving complete stable remission, pharmacological remission, or minimal manifestations for at least 1 year. An unfavorable outcome included the categories improved, unchanged, worse, exacerbation, or died of MG. To be classified as having a favorable outcome, patients must not have received intravenous immunoglobulins (IVIg) or undergone plasma exchange within the past year.

Patients with comorbid autoimmune diseases were compared to those without other autoimmune diseases. Additionally, comparisons were made between male and female patients with comorbid autoimmune diseases, as well as between patients with and without autoimmune thyroid diseases.

This study was conducted in accordance with the ethical standards set forth in the Declaration of Helsinki. It received approval from the ethics committee of Bursa Uludag University (approval number 2023/27–28). As this is a retrospective study and it is not registered in the clinical trials registry. The requirement for direct informed consent was waived due to the anonymized nature of the data. We believe that the lack of registration in the clinical trials registry does not significantly impact the study’s results or conclusions.

To assess the impact of ADs on the clinical course of MG, multivariable logistic regression analyses were performed. The primary outcome was prognosis, classified as favorable versus unfavorable. A secondary analysis was conducted using the worst MGFA classification during disease, dichotomized as severe (≥3) or mild (<3). In both models, sex and age at onset were included as covariates to control for potential confounding. Given the imbalanced distribution between groups (41 vs. 261), Firth’s penalized logistic regression was additionally performed to reduce potential bias in parameter estimates using the *logistf* R package [9].

Statistical Package for the Social Sciences (IBM SPSS Statistics, New York, USA) 28.0 and R 4.4.3 software was used for statistical evaluations, and p < 0.05 was accepted as the limit of significance. The Pearson and Fisher’s exact tests chi-square test was performed for the comparison of categorical variables between the groups. For the comparison of quantitative variables between two independent groups comparisons, Mann– Whitney U Test was used.

## 3. Results

### 3.1 Study population

Out of the initial cohort of 350 patients, 12 were excluded for not meeting the inclusion criteria, and an additional 26 were excluded due to non-relevant data, including a history of autoimmunity in the family. As a result, 302 patients were included in the study, consisting of 41 patients with comorbid autoimmune diseases and 261 without any other autoimmune diseases. Table 1 presents a summary of the clinical characteristics of the enrolled patients.

**Table 1:** Demographic and clinical characteristics of included MG cases.

		Included cohort (N = 302)
Age at onset, years [median (Q1-Q3)]		55.00 (34.00-64.25)
Follow-up duration, years [median (Q1-Q3)]		6.00 (4.0-12.00)
Sex	Male	156 (51.7%)
	Female	146 (48.3%)
Antibody profile	AChR	232 (76.8%)
	MuSK	16 (22.9%; 5.3% of total)^¥^
	Double seronegative	54 (17.9%)
Phenotype	Ocular onset	155 (51.3%)
	Generalized onset	147 (48.7%)
Comorbid autoimmune disease		41 (13.6%)

¥ Anti-MuSK testing was performed only in AChR-negative patients; values are shown as n (%, % of total cohort). Q1, first quartile; Q3, third quartile.

### 3.2 Prevalence of Comorbid ADs in MG patients

A total of 41 patients (29 females and 12 males, 13.6%) with MG had at least one comorbid AD. Among the 302 MG patients, thyroid disease was the most common comorbid AD, observed in 32 patients (10.6%). Hashimoto’s thyroiditis was the most frequent thyroid disorder (n = 22, 7.3%), followed by Graves’ disease (n = 10, 3.3%). Other comorbid ADs included rheumatoid arthritis (n = 4, 1.3%), bullous pemphigoid (n = 1, 0.3%), Sjögren’s syndrome (n = 1, 0.3%), systemic lupus erythematosus (n = 1, 0.3%), lichen planus (n = 1, 0.3%), psoriasis (n = 1, 0.3%), vitiligo (n = 1, 0.3%), transverse myelitis (n = 1, 0.3%), autoimmune hepatitis (n = 1, 0.3%), chronic urticaria (n = 1, 0.3%), autoimmune hypoparathyroidism (n = 1, 0.3%), multiple sclerosis (n = 1, 0.3%). Additionally, 5 patients (1.7%) had more than one comorbid autoimmune disease. Of the 41 patients with comorbid ADs, 32 were diagnosed with other AD before the onset of MG, while 9 patients developed other AD after MG diagnosis. Among those diagnosed with AD before MG diagnosis, 8 patients had received immunosuppressive treatment for their non-MG ADs. These treatments included methotrexate, cyclosporine, azathioprine, mycophenolate mofetil, corticosteroids, and tacrolimus. Autoimmune conditions treated with immunosuppressive agents included rheumatoid arthritis, ulcerative colitis, lichen planus, vitiligo, and chronic urticaria.

### 3.3 Comparison of MG patients with and without comorbid ADs

Patients with comorbid autoimmune diseases demonstrated a higher frequency of autoimmune conditions among their family members compared to those without comorbidities. Autoimmune diseases were also more prevalent in female patients. No statistically significant differences were identified between the two groups in terms of AChR, MuSK or seronegative myasthenia rates (p > 0.05). Additionally, the onset clinical presentations of the patients did not differ significantly between the groups (p > 0.05). The rates of thymectomy and the prevalence of thymoma in thymectomized patients were comparable between the two groups (p > 0.05). Similarly, no significant differences were found in myasthenic exacerbation rates, intensive care unit admissions, outcomes, or the worst MGFA classifications between patients with and without comorbid autoimmune diseases (p > 0.05). Treatment regimens were also similar across the two groups, with no statistically significant differences observed (p > 0.05) (Table 2).

**Table 2:** Comparison of the patients with comorbid ADs with patients without comorbid ADs.

	Comorbid AD (N = 41)	Without comorbid AD (N = 261)	p
Age at diagnosis, years [median(Q1-Q3)]	52 (33.50-62.00)	56 (34.00-65.50)	0.385
Follow-up duration, years [median(Q1-Q3)]	7 (4.00-12.00)	6 (4.00-12.00)	0.935
Late-onset MG (>50), n (%)	23 (56.1%)	149 (57.1%)	0.518
Sex (M/F), n (%)	12/29 (29.3%/70.7%)	134/127 (51.3%/48.7%)	**0.014**
Family history of AD, n(%)	9 (22.0%)	18 (6.9%)	**0.005**
Ocular Onset, n (%)	17 (41.5%)	138 (52.9%)	0.117
**Antibody Status n (%)**			
Anti-AChR-Ab seropositivity	33 (80.5%)	199 (76.2%)	0.690
Anti- MuSK-Ab seropositivity	3 (37.5%; 7.3% of total)^¥^	13 (21.0%; 5% of total)^¥^	0.373
Double seronegativity	5 (12.2%)	49 (18.8%)	0.422
**Outcomes, n (%)**			
Myasthenic exacerbations	19 (46.3%)	99 (37.9%)	0.393
Hospitalization in ICU	6 (14.6%)	34 (13%)	0.973
**Worst MGFA score**			
MGFA 1	3(7.3%)	42(16.1%)	0.130
MGFA 2	9(22.0%)	83(31.8%)	
MGFA 3	20(48.8%)	78(29.9%)	
MGFA 4	4(9.8%)	31(11.9%)	
MGFA5	5(15.6%)	27(10.3%)	
Unfavorable outcome	1(2.4%)	14 (5.4%)	0.370
Thymectomy, n (%)*	14(34.1%)	72 (27.6%)	0.497
Presence of thymoma, n (%)	7 (50%)	31 (42.5%)	0.821
**Previous treatments, n (%)**			
Pyridostigmine	41(100%)	259(99.2%)	0.747
Steroids	34(82.9%)	194 (74.3%)	0.320
Azathioprine	29(70.7%)	144(55.2%)	0.089
Mycophenolate mofetyl	5(12.2%)	29(11.1%)	0.504
Rituximab	8 (19.5%)	29 (11.1%)	0.204
Methotrexate	8(19.5%)	38(14.6%)	0.557
Eculizumab	1 (2.4%)	5 (1.9%)	0.587

*in a total of 86 patients who underwent thymectomy, ^¥^Anti-MuSK testing was performed only in AChR-negative patients; values are shown as n (%, % of total cohort). Q1, first quartile; Q3, third quartile. Age at diagnosis and Follow-up duration (quantitative variables) were compared with Mann-Whitney U test. All other variables (categorical) were compared with the Pearson or Fisher’s exact chi-square test.

In the multivariable logistic regression analysis assessing the effect of autoimmune disease on prognosis (favorable vs. unfavorable outcome), after controlling for sex and age at onset, the overall model was not statistically significant (Omnibus test, p = 0.580). Thus, autoimmune disease was not identified as an independent predictor of prognosis (S1 Table). In contrast, in a separate model using the worst MGFA classification (≥3 vs. < 3) as the dependent variable, sex emerged as a significant predictor, with female gender increasing the risk of severe MG (OR = 1.82, 95% CI = 1.13–2.94, p = 0.014). In this model, autoimmune disease and age at onset were not significant predictors (p = 0.070 and p = 0.612, respectively). Results were consistent in the Firth’s penalized logistic regression, supporting the robustness of the estimates (S2 Table).

### 3.4 Comparison of male and female MG patients with comorbid ADs

Given the higher prevalence of autoimmune diseases in female patients, a comparison was made between male and female patients with comorbid autoimmune diseases. Generalized onset was more frequently observed in male patients, whereas myasthenic exacerbations were more common in female patients. However, no statistically significant differences were found between the two groups in terms of the worst MGFA classification or overall outcomes (p > 0.05).

The age of onset, thymectomy rates, and the prevalence of thymoma in patients who underwent thymectomy were similar between the male and female MG patients (p > 0.05).

Notably, the use of rituximab was significantly more frequent in female patients compared to male patients (p < 0.05). In contrast, the use of other immunosuppressive therapies was found to be similar across both groups (p > 0.05) (Table 3).

**Table 3:** Comparison of the patients with comorbid ADs based on gender.

	Men with comorbid AD (N = 12)	Women with comorbid AD (N = 29)	P
Age at diagnosis, years [median (Q1-Q3)]	52.50 (38.00-62.75)	51.00 (28.50-62.00)	0.566
Follow-up duration, years [median (Q1-Q3)]	5.00 (3.25-7.00)	7.00 (4.00-17.00)	0.101
Late-onset MG (>50), n (%)	7 (58.3%)	16 (55.2%)	0.566
Ocular Onset, n (%)	10 (83.3%)	14 (48.3%)	**0.039**
Family history of AD, n (%)	2 (16.7%)	7(24.1%)	0.470
**Antibody Status, n (%)**			
Anti-AChR-Ab seropositivity	9 (75.0%)	24 (82.8%)	0.431
Anti- MuSK-Ab seropositivity	1 (33.3%; 8.3% of total)^¥^	2 (40%; 6.9% of total)^¥^	0.657
Double seronegativity	2 (16.7%)	3 (10.3%)	0.461
**Outcomes, n (%)**			
Myasthenic exacerbations	2(16.7%)	17(58.6%)	**0.035**
Hospitalization in ICU	0 (0%)	6 (20.7%)	0.106
**Worst MGFA score**			
MGFA 1	1(8.3%)	2(6.9%)	0.938
MGFA 2	2(16.7%)	7(24.1%)	
MGFA 3	7(58.3%)	13(44.8%)	
MGFA 4	1(8.1%)	4(13.8%)	
MGFA5	1(8.3%)	4(13.8%)	
Unfavorable outcome	0(0%)	1 (3.4%)	0.707
Thymectomy	3(25.0%)	11(37.9%)	0.338
Presence of thymoma, n (%)*	2(66.7%)	5(45.5%)	0.500
Previous treatments, n (%)			
Pyridostigmine	12(100%)	29(100%)	
Steroids	11(91.7%)	23(79.3%)	0.323
Azathioprine	7(758.3%)	22(75.9%)	0.226
Mycophenolate mofetyl	1(8.3%)	4(13.8%)	0.539
Rituximab	0 (0%)	8(27.6%)	**0.045**
Methotrexate	3(25.0%)	5(17.2%)	0.431
Eculizumab	0 (0%)	1 (3.4%)	0.707

*in a total of 14 patients who underwent thymectomy, ^¥^Anti-MuSK testing was performed only in AChR-negative patients; values are shown as n (%, % of total cohort). Q1, first quartile; Q3, third quartile.Age at diagnosis and Follow-up duration (quantitative variables) were compared with Mann-Whitney U test. All other variables (categorical) were compared with the Pearson or Fisher’s exact chi-square test.

We considered whether thymectomy was a risk factor for AIDs. Among the patients with autoimmune diseases and thymectomy, a total of 14 patients were identified.

### 3.5 Prognosis of myasthenia gravis patients with and without autoimmune thyroid disease

When autoimmune thyroid diseases were the sole comorbidity and patients with multiple autoimmune conditions were excluded, there were no notable differences in myasthenic exacerbation rates, intensive care unit admissions, overall outcomes or the worst MGFA classifications between MG patients with and without autoimmune thyroid disease (p > 0.05). Likewise, treatment approaches were comparable between the two groups, with no statistically significant variations observed (p > 0.05).

## 4. Discussion

This study investigated the clinical characteristics and outcomes of MG patients with and without comorbid ADs. Our key findings include: (1) approximately 13.6% of MG patients have at least one comorbid AD; (2) ADs occur more frequently in female MG patients compared to male MG patients; (3) autoimmune thyroid diseases (AITD) are the most common comorbid ADs in our cohort; and (4) the presence of comorbid ADs does not appear to worsen the prognosis of MG significantly.

### 4.1 Clinical outcomes

In terms of clinical outcomes, we found no significant differences between MG patients with and without comorbid ADs regarding myasthenic exacerbation rates, ICU admissions, overall outcomes, or the worst MGFA classifications. These findings suggest that comorbid autoimmune diseases may not significantly impact MG severity or prognosis. This result aligns with certain studies indicating that the presence of ADs does not uniformly alter MG’s clinical course [6,10]. However, it contrasts with other reports suggesting a more severe disease course in MG patients with ADs, potentially due to an amplified autoimmune response or increased inflammatory burden [3,11]. Such discrepancies may result from differences in study populations and races, methodologies, or the specific types of comorbid ADs included. Future studies with larger, more diverse cohorts and standardized assessments of autoimmune comorbidities are warranted to clarify these associations.

In our cohort, the majority of autoimmune diseases were diagnosed before the onset of MG, and a notable subset of these patients had received immunosuppressive therapy for their pre-existing autoimmune conditions. This temporal relationship may suggest that MG can emerge in the context of ongoing systemic immune dysregulation. Although eight patients had been exposed to immunosuppressive agents prior to MG onset, the available data were not sufficient to determine whether these treatments had any modifying effect on MG development or severity. The retrospective design and small sample size limit our ability to draw causal inferences. Nevertheless, these findings highlight the complexity of immune system interactions in patients with multiple autoimmune diseases and underscore the importance of longitudinal monitoring in this population.

### 4.2 Gender differences in MG with comorbid ADs

When comparing female and male patients with comorbid ADs, we observed that myasthenic exacerbations were more frequent in female patients. Additionally, rituximab use was significantly higher in females compared to their male counterparts. Several factors may explain these observations. Autoimmune diseases are known to be more prevalent in females, possibly reflecting a more pronounced autoimmune response in this group. Hormonal and immunological differences may also contribute to a more severe clinical course in females with MG and comorbid ADs, necessitating more aggressive treatment strategies [12,13]. Indeed, prior studies have suggested that MG tends to manifest as a more severe disease in females [14,15]. However, further research is needed to confirm whether these findings are specific to certain autoimmune comorbidities or influenced by other factors such as treatment variations or healthcare access disparities.

### 4.3 Autoimmune thyroid diseases in MG

The AITD was the most common comorbid AD in our cohort, with a prevalence of 10.6%, consistent with a previous meta-analysis [16] but lower compared to certain other studies [17,18]. This variation might be attributed to differences in diagnostic criteria.

In our study, however, MG patients with AITD did not show significant differences in disease onset, prognosis, or rates of myasthenic exacerbations compared to those without AITD. These findings suggest that AITD may not universally influence MG severity. In the literature, MG patients with AITD are often reported to have a milder clinical course, characterized by more frequent ocular involvement and lower susceptibility to myasthenic crises. This phenomenon is thought to result from immunological cross-reactivity between thyroid and eye muscle autoantigens [18–20].

It is also worth noting that thyroid function and autoimmunity were evaluated based on endocrinology follow-ups and clinical records so standardized and comprehensive assessments could provide more definitive insights into the relationship between AITD and MG.

### 4.4 Limitations

This study has certain limitations. The retrospective design may have introduced potential biases in data collection. Additionally, the relatively small sample size could have reduced the statistical power to detect subtle differences between the groups. Moreover, given the clinical heterogeneity of autoimmune diseases, our results may not fully capture the potential differences between individual conditions.

## 5. Conclusion

In conclusion, our study shows that comorbid autoimmune diseases do not significantly affect the clinical presentation, treatment response, or outcomes in MG. Autoimmune diseases were more common in female patients, who also experienced more frequent myasthenic exacerbations and higher rituximab usage, warranting further investigation. Early recognition of comorbidities remains crucial for optimal management. Larger, prospective studies are needed to confirm these findings and explore their implications.

## Supporting information

S1 Table Multivariable logistic regression analysis for predictors of unfavorable outcome. (DOCX)

S2 Table Multivariable logistic regression and Firth’s penalized logistic regression results for predictors of worst MGFA classification. (DOCX)
