# Supplementary material for: Myasthenia gravis and autoimmune overlap: Prognostic insight
Source: PLoS One. 2025 Oct 23;20(10):e0334434. doi: 10.1371/journal.pone.0334434 (PMC12548888; doi:10.1371/journal.pone.0334434)
Supplement: S1 Table — (DOCX) [file pone.0334434.s001.docx]

S1 Table. Multivariable logistic regression analysis for predictors of unfavorable outcome

| Variable | Logistic regression analysis | |
| --- | --- | --- |
|  | p-value | OR (95% CI) |
| Sex  (RC: males) | 0.348 | 1.69 (0.57–5.02) |
| Onset age  (years) | 0.454 | 1.01 (0.98–1.04) |
| Autoimmune disease (RC: absent) | 0.388 | 0.402 (0.05–3.19) |

Omnibus test for the model: p=0.580

OR: Odds ratio, CI: Confidence interval, RC: Reference category
