# Supplementary material for: Myasthenia gravis and autoimmune overlap: Prognostic insight
Source: PLoS One. 2025 Oct 23;20(10):e0334434. doi: 10.1371/journal.pone.0334434 (PMC12548888; doi:10.1371/journal.pone.0334434)
Supplement: S2 Table — (DOCX) [file pone.0334434.s002.docx]

S2 Table. Multivariable logistic regression and Firth’s penalized logistic regression results for predictors of worst MGFA classification

| Variable | Logistic regression analysis^1^ | | Firth logistic regression analysis^2^ | |
| --- | --- | --- | --- | --- |
|  | p-value | OR (95% CI) | p-value | OR (95% CI) |
| Sex  (RC: males) | **0.014** | 1.82 (1.13–2.94) | **0.014** | 1.81 (1.13–2.92) |
| Onset age  (years) | 0.612 | 1.00 (0.98–1.01) | **0.614** | 1.00 (0.98–1.01) |
| Autoimmune disease (RC: absent) | 0.070 | 1.96 (0.95–4.06) | **0.068** | 1.91 (0.95–4.02) |

Omnibus test: ^1^p=0.006, ^2^p=0.007

OR: Odds ratio, CI: Confidence interval, RC: Reference category
